# Supplementary material for: Serum Neurofilament Light Chain and Glial Fibrillary Acidic Protein as Potential Diagnostic Biomarkers in Autism Spectrum Disorders: A Preliminary Study
Source: Int J Mol Sci. 2023 Feb 3;24(3):3057. doi: 10.3390/ijms24033057 (PMC9917818; doi:10.3390/ijms24033057)
Supplement: Supplementary file 1 [file ijms-24-03057-s001.zip › ijms-2149084-supplementary.pdf]

International Journal of *Molecular Sciences*

Supplementary Materials

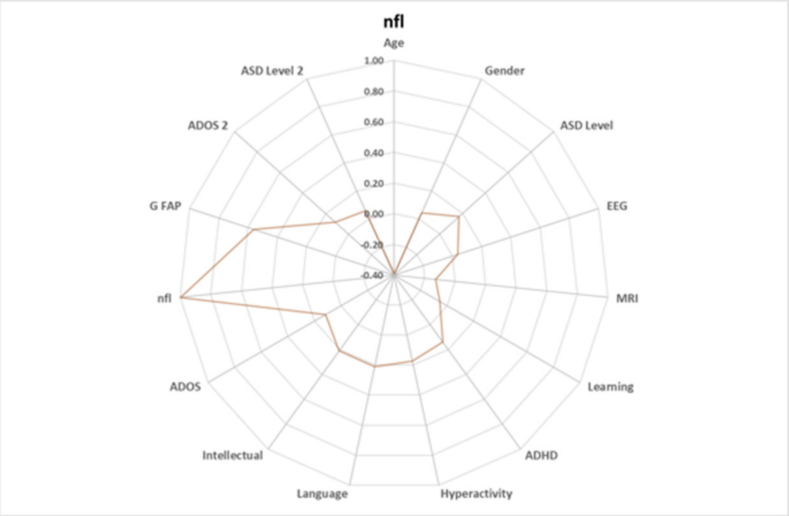

Figure S1. Star plot of the correlation test results of Nfl levels with clinical features in ASD sample.

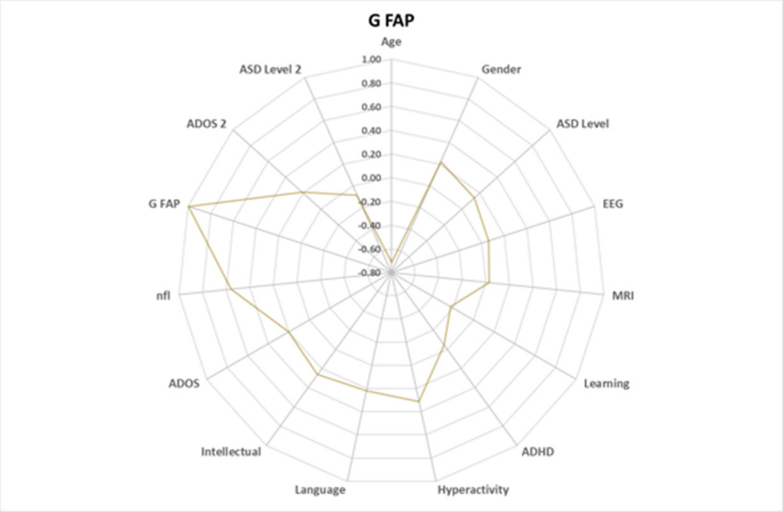

Figure S2. Star plot of the correlation test results of GFAP levels with clinical features in ASD sample.
